# The relationship between preoperative anemia and length of hospital stay among patients undergoing radical surgery for esophageal carcinoma: a single-centre retrospective study

**DOI:** 10.1186/s12871-023-02235-5

**Published:** 2023-10-01

**Authors:** Zonggui Zheng, Shixuan Peng, Jieping Yang, Weiqi Ke

**Affiliations:** 1 Shantou University Medical College, 22 Xinling Road, Shantou, Guangdong 515041 China; 2 Department of Oncology, Graduate Collaborative Training Base of The First People’s Hospital of Xiangtan City, Hengyang Medical School, University of South China, Hengyang, Hunan 421001 China; 3 Department of Anesthesiology, Royallee Cancer Hospital, No.1, Ciji Road, Huangpu District, Guangzhou, Guangdong 510555 China; 4 Department of Anesthesiology, The First Affiliated Hospital of Shantou University Medical College, 57 Changping Road, Shantou, Guangdong 515041 China

**Keywords:** Radical surgery for esophageal carcinoma, Preoperative anemia, Multivariate linear regression, Length of stay

## Abstract

**Background:**

Although it is unclear if preoperative anemia affects patients undergoing radical resection of esophageal cancer, it does increase the length of stay (LOS) for surgical patients. Accordingly, the purpose of this study was to investigate if, after adjusting for other covariates, anemia was independently associated with LOS in people undergoing radical resection of esophageal cancer.

**Methods:**

The retrospective cohort study included 680 patients undergoing radical esophageal cancer surgery between January 2010 and December 2020. Preoperative anemia was the targeted independent variable, while LOS was the target independent variable. Demographics, comorbidities, laboratory tests, surgery and anesthesia, postoperative outcomes, and complications were collected. Multivariate linear analyses were performed for variables that might influence preoperative anemia and LOS selection. Subgroup analysis using hierarchical variables was then used to test the potential relationship.

**Results:**

The 647 individuals that were randomly chosen had an average age of 61.06 ± 8.16 years, and 77.43% of them were male. The prevalence of anemia was 36.6%. All patients recruited had an average length of stay (LOS) of 26.31 ± 13.19 days, 25.40 ± 11.44 days for patients who had no preoperative anemia, and 27.89 ± 15.66 days for patients who had preoperative anemia, *p* < 0.05. After adjusting for covariates, the results of fully adjusted linear regression revealed that preoperative anemia was significantly associated with LOS (β = 2.04, 95%CI (0.13, 3.96) ), *p* < 0.05. The results of the subgroup analysis were basically accurate and steady. Regardless of gender, same outcomes were seen when preoperative anemia was defined as a Hb level < 13 g/dL (β = 2.29, 95%CI (0.33, 4.25) ), *p* < 0.05. In addition, the LOS was shortened with the increase of preoperative hemoglobin (Hb) (β= -0.81, 95%CI (-1.46, -0.1) ), *p* < 0.05.

**Conclusion:**

Preoperative anemia is typical in Chinese patients undergoing radical esophageal cancer resection and is independently associated with prolonged LOS.

## Introduction

Esophageal carcinoma is the eighth most prevalent cancer worldwide and has the sixth-highest death risk due to cancer [1]. The only curative treatment for resectable oesophageal cancer is considered to be surgical resection with radical lymphadenectomy, often following neoadjuvant chemotherapy or chemoradiotherapy [2–4].

Anemia is classified according to WHO definitions [5]. Approximately 30% of cancer patients have anemia, which is a disease that is known to be widespread in this population [6, 7]. Esophageal cancer patients are not an exception. Most patients before surgery have varying degrees of anemia [8]. Surgical patients who have preexisting anemia prior to intestinal surgery are at higher risk for negative surgical outcomes, such as an increased likelihood of blood transfusions and longer hospital stays [9–11]. A prospective observational study was carried out in South Africa, which included patients undergoing inpatient surgeries unrelated to cardiology or obstetrics in 50 hospitals. The study found that patients with preexisting anemia before surgery had a notably prolonged length of hospital stay [12]. Based on previous reports, individuals with anemia have a higher likelihood of requiring blood transfusions, which can increase the occurrence of postoperative complications and wound infections. This can consequently impede early recovery and prolong the length of hospital stay for these patients [13–15]. In most surgical procedures, preoperative anemia is associated with a longer hospital stay. However, prolonged hospitalization may not be advantageous as it can lead to increased inefficiency in the use of medical resources, higher medical expenses, and an increased burden of health care on society [16].

Preoperative anemia and LOS in patients having radical esophageal cancer resection are known to be correlated, although the exact nature of this association is unclear. The purpose of this study was to determine if anemia is independently associated with LOS in patients receiving radical resection of esophageal cancer.

## Participants and methods

### Study design

We conducted a retrospective cohort study to find out how preoperative anemia and LOS are related. Preoperative anemia was the targeted independent variable, while LOS was the target independent variable. Meanwhile, Hb obtained at baseline was analyzed with LOS as a target independent variable in order to further prove the association between LOS and preoperative anemia.

### Study population

**Fig. 1:**
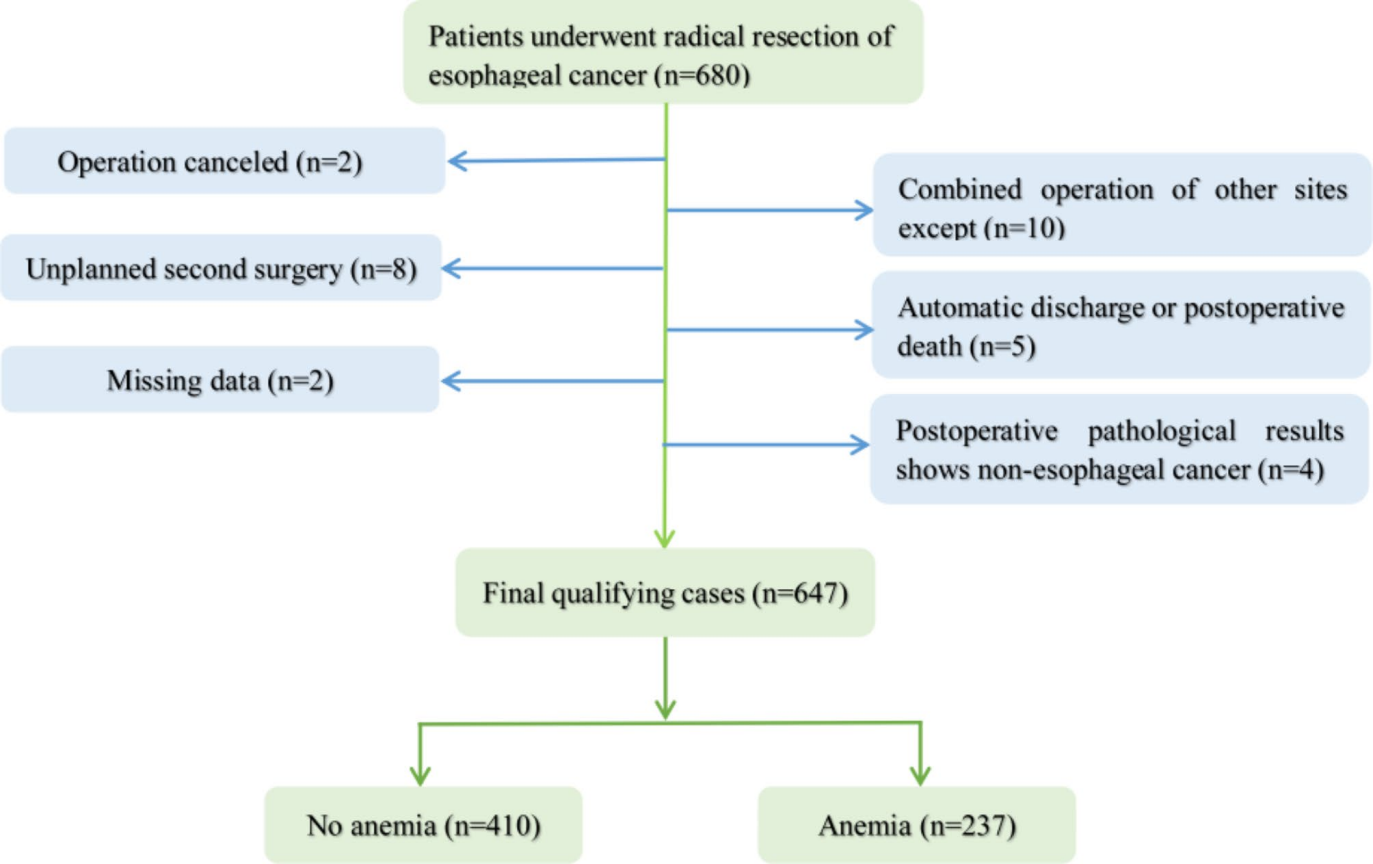
Flowchart of patient selection

All patients who received radical resection of esophageal cancer between January 2010 and December 2020 (n = 680) were included. The following patients were excluded: (1) Unplanned second surgery (n = 8); (2) The combined operation of other sites except for the esophagus (n = 10); (3) Automatic discharge or postoperative death (n = 5); (4) Operation canceled (n = 4); (5) Postoperative pathological results showed non-esophageal cancer (n = 4); (6) Missing data (n = 2). The final number of cases was 647. The average age of the 647 individuals was 61.06 ± 8.16 years, and 77.43% of them were male. The percentages of esophageal cancer surgical patients with hypertension and pulmonary disease are 15.77% and 23.15%, respectively. Postoperative lung infections occurred in 32.77% of the population of patients undergoing radical surgery for esophageal carcinoma. (Fig. 1) To protect patients’ privacy, we did not include information about any individuals who may be identified in our data. The electronic medical record system at the hospital was used to compile the data. Because this was a retrospective cohort research, informed permission from the participants is not necessary. This research was authorized by the hospital’s institutional review board (NO. B-2021-249).

### Variables

We obtained data from the clinical data management system of a medical center in China on patients undergoing radical resection of esophageal cancer, including the continuous and categorical variables we needed (Table 1).

We considered whether patients had preoperative anemia as a categorical variable. Preoperative anemia was defined as Hb < 130 g/ L in males or Hb < 120 g/L in females according to WHO gender-based criteria [17].

From the date of admission to the date of hospital discharge to the patient’s home setting, we considered LOS as a continuous variable.

From the database, we selected the following categories of variables for this study: The following information is provided: (1) demographic information; (2) variables that, according to previous research, might impact anemia or LOS; (3) comorbidities; and (4) clinical experience. Consequently, the fully-adjusted model was built using the following variables: (1) continuous variable: age; preoperative hemoglobin (Hb); serum creatinine (SCr); white blood cells (WBC); prothrombin time (PT); serum sodium ion (Na^+^); serum potassium ion (K^+^); activated partial prothrombin time (APTT ); duration of surgery; blood loss; fluid infusion; (2) categorical variables: gender; lung disease; diabetes; heart disease; central nervous system; hypertension; preoperative chemotherapy; anesthesia; operation form; blood transfusion; postoperative admission to intensive care unit (ICU); respiratory failure; lung infection; anastomotic fistula; cardiovascular complication; American Society of Anesthesiologists (ASA) status.

### Statistical analysis

We divided the patients into two groups based on whether they had preoperative anemia or not, and performed a correlation analysis to determine whether preoperative anemia was associated with length of stay (LOS) in patients undergoing radical esophageal cancer surgery. Although continuous variables were provided as mean, standard deviation (SD), or median while categorical variables are shown as numbers and percentages (skewed distribution). The χ^2^ (categorical variables), Student’s t-test (normal distribution), and Mann-Whitney U test (skewed distribution) were used to compare the preoperative anemia group with the nonpreoperative anemia group. To investigate the relationship between preoperative anemia and LOS, we tested preoperative Hb (g/dL) and LOS (days) using univariate and multivariate linear regression models with three different models, crude models without adjusting for any variable; model 1, for socio-demographic data only (age and sex); and model 2, for socio-demographic data and other covariates in Table 1 [18]. Because in the presence of a nonlinear relationship between preoperative Hb and LOS, generalized additive models and smoothed curve fitting (penalized spline method) were used to solve the nonlinearity problem. If nonlinearity was found, we created a two-slice binary logistic regression on both sides of the inflection point after first calculating the inflection point using recursive techniques. After all, the log-likelihood ratio test primarily determines which model is better suited to establish the link between the target independent variable and the outcome variable. Stratified linear regression models were used for subgroup analysis. In order to do an interaction test on continuous variables, we first transformed the data to categorical variables based on the clinical cut-off point or tertile. Following tests for effect modification of subgroup indicators, the likelihood ratio test was conducted [19]. The statistical software programs R (http://www.r-project.org, The R Foundation) and EmpowerStats (http://www.empowerstats.com, X&Y Solutions, Inc., Boston, MA) were used for all analyses. Statistical significance was defined as *p*-values less than 0.05 (two-sided) [20].

## Results

### Baseline characteristics of selected participants

Following the screening of participants using the inclusion and exclusion criteria, 647 participants were chosen for the final data analysis (see Fig. 1 for a flow chart). Based on whether the patients had preoperative anemia, the baseline characteristics of these chosen participants are displayed in Table 1. Overall, the 647 participants who were ultimately selected had an average age of 61.06 ± 8.16 years, and around 77.43% of them were male. The non-preoperative anemia group included 410 patients, while the preoperative anemia group included 237 patients. There were no statistically significant differences in diabetes, pulmonary disease, hypertension, central nervous system, Na^+^, K^+^, SCr, PT, ASA, anesthesia, gender, heart disease, surgical form, duration of surgery, blood loss, transfusion, postoperative admission to ICU, respiratory failure, pulmonary infection, anastomotic fistula, and cardiovascular complications between different groups (all *p* > 0.05). The mean age of the preoperative anemia group (62.31 ± 7.79) was higher than that of the non-preoperative anemia group (60.34 ± 8.30) (*p* < 0.05). Of the 647 patients, 142(21.95%) received adjuvant chemotherapy. The number of patients receiving preoperative chemotherapy in the preoperative anemia group (99 (41.77%) was higher than that in the non-preoperative anemia group (43 (10.49%) (*p* < 0.05). The mean preoperative Hb concentration in the group without preoperative anemia (13.95 ± 0.97) was higher than that in the group with preoperative anemia (11.59 ± 1.04) (*p* < 0.05). The perioperative transfusion rate of patients with esophageal cancer was 127(19.63%). Participants in the preoperative anaemia group showed a higher mean age (*p* < 0.05).The transfusion rate of the preoperative anemia group was 69 (29.11%) higher than that of the non-preoperative anemia group, 58(14.15%) (*p* < 0.05). The overall LOS was (26.31 ± 13.19) days. Hb and blood transfusion in both groups, with *p*-values less than 0.001, were statistically significant. In addition, compared with the non-preoperative anemia group (25.40 ± 11.44), the LOS of the preoperative anemia group (27.89 ± 15.66) was longer (*p* < 0.05). In addition, there was a difference in WBC and APTT between the two groups (*p* < 0.05).

**Table 1:** Baseline characteristics of participants. (N = 647)

Variable	Total	No anemia	Anemia	*p*-value
**N**	647	410	237	
Age, (year)	61.06 ± 8.16	60.34 ± 8.30	62.31 ± 7.79	0.003
Gender, n (%)				0.492
Male	501 (77.43%)	321 (78.29%)	180 (75.95%)	
Female	146 (22.57%)	89 (21.71%)	57 (24.05%)	
Comorbidities, n (%)				
Hypertension	102 (15.77%)	64 (15.61%)	38 (16.03%)	0.887
Diabetes	47 (7.26%)	27 (6.59%)	20 (8.44%)	0.382
Heart disease	67 (10.36%)	44 (10.73%)	23 (9.70%)	0.680
Central nervous system	14 (2.16%)	9 (2.20%)	5 (2.11%)	0.943
Lung disease	150 (23.18%)	87 (21.22%)	63 (26.58%)	0.119
Laboratory examination				
Hb, (g/dL)	13.09 ± 1.51	13.95 ± 0.97	11.59 ± 1.04	< 0.001
WBC, (×10^9/L)	7.13 ± 2.14	7.31 ± 1.95	6.82 ± 2.40	0.005
Na^+^, (mmol/L)	139.18 ± 3.14	139.27 ± 3.23	139.02 ± 2.97	0.343
K^+^, (mmol/L)	4.00 ± 0.37	4.01 ± 0.35	3.99 ± 0.40	0.476
SCr, (mol/L)	90.51 ± 23.12	90.56 ± 19.69	90.41 ± 28.14	0.937
PT, (s)	11.02 ± 0.87	10.99 ± 0.85	11.06 ± 0.91	0.305
APTT, (s)	27.54 ± 4.86	27.25 ± 4.70	28.04 ± 5.08	0.047
Preoperative chemotherapy	142 (21.95%)	43 (10.49%)	99 (41.77%)	< 0.001
ASA ≥ 3	53 (8.19%)	28 (6.83%)	25 (10.55%)	0.097
Anesthesia				0.445
General + epidural	462 (71.41%)	297 (72.44%)	165 (69.62%)	
General	185 (28.59%)	113 (27.56%)	72 (30.38%)	
Operation form				0.856
Minimally invasive	300 (46.37%)	189 (46.10%)	111 (46.84%)	
Open	347 (53.63%)	221 (53.90%)	126 (53.16%)	
Operation time, (min)	239.13 ± 56.72	239.29 ± 61.03	238.87 ± 48.48	0.928
Blood loss, (ml)	198.61 ± 157.74	198.07 ± 168.76	199.54 ± 136.92	0.881
Fluid infusion, (ml)	2556.26 ± 604.97	2572.93 ± 608.48	2527.43 ± 599.05	0.357
Blood transfusion	127 (19.63%)	58 (14.15%)	69 (29.11%)	< 0.001
Postoperative admission to ICU	42 (6.49%)	23 (5.61%)	19 (8.02%)	0.231
Postoperative complications				
Respiratory failure	24 (3.71%)	15 (3.66%)	9 (3.80%)	0.928
Lung infection	212 (32.77%)	129 (31.46%)	83 (35.02%)	0.353
Anastomotic fistula	42 (6.49%)	28 (6.83%)	14 (5.91%)	0.646
Cardiovascular complication	33 (5.10%)	20 (4.88%)	13 (5.49%)	0.735
LOS, (days)	26.31 ± 13.19	25.40 ± 11.44	27.89 ± 15.66	0.021

Data are expressed as mean ± standard deviation, or number (per-centage)

**Abbreviations**: LOS, length of stay; Hb, hemoglobin; WBC, white blood cells; Na^+^, Serum sodium ion; K^+^, Serum potassium ion; SCr, Serum creatinine; PT, prothrombin time; APTT, activated partial thromboplastin time; ASA, American Society of Anesthesiologist; RBC, red blood cell; ICU, Intensive Care Unit

### Univariate analysis

Table 2 contains a summary of the findings of our univariate analyses. By using univariate linear regression, we found that lung disease, heart disease, central nervous system, gender, WBC, Na^+^, K^+^, SCr, PT, APTT, preoperative chemotherapy, ASA, anesthesia, blood loss, and fluid infusion were not associated with LOS. We also found that open operations (-2.36, -4.39- -0.33) were negatively associated with LOS. In contrast, univariate analysis revealed that preoperative anemia (2.49, 0.39–4.59 vs. ref); Age, (years)(0.15, 0.03–0.28); age ≥ 60 years (2.45, 0.40–4.50 vs. ref); hypertension (3.81, 1.04–6.59 vs. ref); diabetes (6.12, 2.23–10.01 vs. ref); preoperative Hb,(g/dL) (-0.93, -1.60- -0.26); preoperative Hb, (g/dL) < 13 (2.41, 0.36–4.45)vs ref); operation time ≥ 240 min (2.37, 0.34–4.40 vs. ref); perioperative RBC infusion (3.80, 1.26- 6.34vs ref); postoperative admission to ICU (11.46, 7.43-15.49vs ref); respiratory failure (6.04, 0.68-11.40vs ref); lung infection (4.17 ,2.02-6.31vs ref); anastomotic fistula ( 26.94, 23.38-30.51vs ref) were positively correlated with LOS.

**Table 2:** Univariate analysis for LOS(Days)

	Statistics	LOS(days) β(95%CI)	P-value
Anemia	237 (36.63%)	2.49 (0.39, 4.59)	0.0205
Age, years	61.06 ± 8.16	0.15 (0.03, 0.28)	0.0164
Age ≥ 60 years	373 (57.65%)	2.45 (0.40, 4.50)	0.0194
Gender			0.6947
Female	146 (22.57)	Reference	
Male	501 (77.43%)	-0.49 (-2.92, 1.95)	
Previous disease			
Hypertension	102 (15.77%)	3.81 (1.04, 6.59)	0.0073
Diabetes	47 (7.26%)	6.12 (2.23, 10.01)	0.0021
Heart disease	67 (10.36%)	1.12 (-2.21, 4.46)	0.5101
Central nervous system	14 (2.16%)	1.58 (-5.41, 8.57)	0.6571
Lung disease	150 (23.18%)	2.04 (-0.37, 4.44)	0.0972
Laboratory examination			
Preoperative Hb, (g/dL)	13.09 ± 1.51	-0.93 (-1.60, -0.26)	0.0068
Preoperative Hb, (g/dL) < 13	278 (42.97%)	2.41 (0.36, 4.45)	0.0215
Abnormal WBC	68 (10.51%)	1.76 (-1.55, 5.07)	0.2983
Abnormal Na^+^	61 (9.43%)	1.56 (-1.92, 5.04)	0.3794
Abnormal K^+^	60 (9.27%)	1.09 (-2.41, 4.60)	0.5410
SCr ≥ 100 mol/L	172 (26.58%)	1.01 (-1.29, 3.31)	0.3915
PT ≥ 13s	15 (2.32%)	-5.02 (-11.77, 1.72)	0.1449
APTT ≥ 37s	11 (1.70%)	-1.42 (-9.29, 6.44)	0.7231
Preoperative chemotherapy	142 (21.95%)	-0.24 (-2.70, 2.22)	0.8478
ASA ≥ 3	53 (8.19%)	2.73 (-0.98, 6.43)	0.1493
Anesthesia			
General + epidural	462 (71.41%)	Reference	
General	185 (28.59%)	2.19 (-0.06, 4.43)	0.0564
Operation form			
Minimally invasive	300 (46.37%)	Reference	
Open	347 (53.63%)	-2.36 (-4.39, -0.33)	0.0231
Operation time ≥ 240 min	313 (48.38%)	2.37 (0.34, 4.40)	0.0223
Blood loss > = 200ml	360 (55.64%)	-0.64 (-2.68, 1.41)	0.5420
Fluid infusion(ml) group			
<2000	39 (6.03%)	Reference	
≥2000, < 3000	396 (61.21%)	0.33 (-4.02, 4.67)	0.8824
≥3000	212 (32.77%)	0.80 (-3.71, 5.31)	0.7297
Perioperative RBC infusion	127 (19.63%)	3.80 (1.26, 6.34)	0.0035
Postoperative admission to ICU	42 (6.49%)	11.46 (7.43, 15.49)	< 0.0001
Postoperative complications			
Respiratory failure	24 (3.71%)	6.04 (0.68, 11.40)	0.0275
Lung infection	212 (32.77%)	4.17 (2.02, 6.31)	0.0002
Anastomotic fistula	42 (6.49%)	26.94 (23.38, 30.51)	< 0.0001
Cardiovascular complication	33 (5.10%)	3.86 (-0.75, 8.47)	0.1016

**Abbreviations**: Abbreviations: LOS, length of stay; Hb, hemoglobin; WBC, white blood cells; Na^+^, Serum sodium ion; K^+^, Serum potassium ion; SCr, Serum creatinine; PT, prothrombin time; APTT, activated partial thromboplastin time; ASA, American Society of Anesthesiologist; RBC, red blood cell; ICU, Intensive Care Unit

### Unadjusted and adjusted linear regression results

This study investigated the impact of preoperative anemia on length of hospital stay (LOS) using three different models: univariate, multivariate linear, and logistic regression. The effect sizes (β) and 95% confidence intervals were reported in Tables 3 and 4.

In Table 3, the unadjusted model showed that LOS was significantly higher in the preoperative anemia group compared to the non-preoperative anemia group (β = 2.49, 95%CI 0.39–4.59, *p* < 0.05). This difference persisted even after adjusting for some covariates in model 1 (β = 2.22, 95%CI 0.11–4.33, *p* < 0.05), and all covariates in model 2 (β = 2.04, 95%CI 0.13–3.96, *p* < 0.05).

Table 4 examined the independent effects of preoperative hemoglobin (Hb) on LOS using univariate and multivariate linear regression. In the unadjusted model, each 1 g/dL increase in preoperative Hb corresponded to a statistically significant decrease in LOS (β=-0.93, 95%CI -1.60 to -0.26, *p* < 0.05). These results remained significant after adjusting for some covariates in model 1 (β=-0.84, 95%CI -1.54 to -0.15, *p* < 0.05), and all covariates in model 2 (β=-0.81, 95%CI -1.46 to -0.16, *p* < 0.05).

To confirm these findings, preoperative Hb was also analyzed as a categorical variable (Hb ≥ 13 g/dL or Hb < 13 g/dL). The results were consistent with those in Tables 3 and 4, where the preoperative Hb < 13 g/dL group had significantly higher LOS compared to the preoperative Hb ≥ 13 g/dL group in all models.

**Table 3:** Relationship between preoperative anemia and LOS (Days)

Outcome	LOS(Days) β(95%CI) *p*-value		
	Crude Model	Model I	Model II
Non-anamia	Reference	Reference	Reference
Anamia	2.49 (0.39, 4.59) 0.0205	2.22 (0.11, 4.33) 0.0400	2.04 (0.13, 3.96) 0.0369

**Abbreviations**: LOS, length of stay; CI, confidence interval

Model I adjusted for age; gender

Model II adjusted for age; gender; hypertension; diabetes; heart disease; central nervous system; lung disease; WBC; Na^+^; K^+^; SCr; PT; APTT; preoperative chemotherapy; ASA; anesthesia; operation form; blood transfusion; operation time; blood loss; fluid infusion; postoperative admission to ICU; respiratory failure; lung infection; anastomotic fistula; cardiovascular complication

**Table 4:** Relationship between preoperative Hb (g/dL) and LOS (Days)

Outcome LOS(Days) β(95%CI) *p*-value			
	**Crude Model**	**Model I**	**Model II**
Hb, (g/dL)	-0.93 (-1.60, -0.26) 0.0068	-0.84 (-1.54, -0.15) 0.0181	-0.81 (-1.46, -0.16) 0.0156
Hb, (g/dL) groups			
≥ 13	Reference	Reference	Reference
<13	2.41 (0.36, 4.45) 0.0215	2.15 (0.03, 4.27) 0.0468	2.29 (0.33, 4.25) 0.0224

Abbreviations: LOS, length of stay; CI, confidence interval

Model I adjusted for age; gender

Model II adjusted for age; gender; hypertension; diabetes; heart disease; central nervous system; lung disease; WBC; Na^+^; K^+^; SCr; PT; APTT; preoperative chemotherapy; ASA; anesthesia; operation form; blood transfusion; operation time; blood loss; fluid infusion; postoperative admission to ICU; respiratory failure; lung infection; anastomotic fistula; cardiovascular complication

### The results of linearity of preoperative hb and LOS

**Fig. 2:**
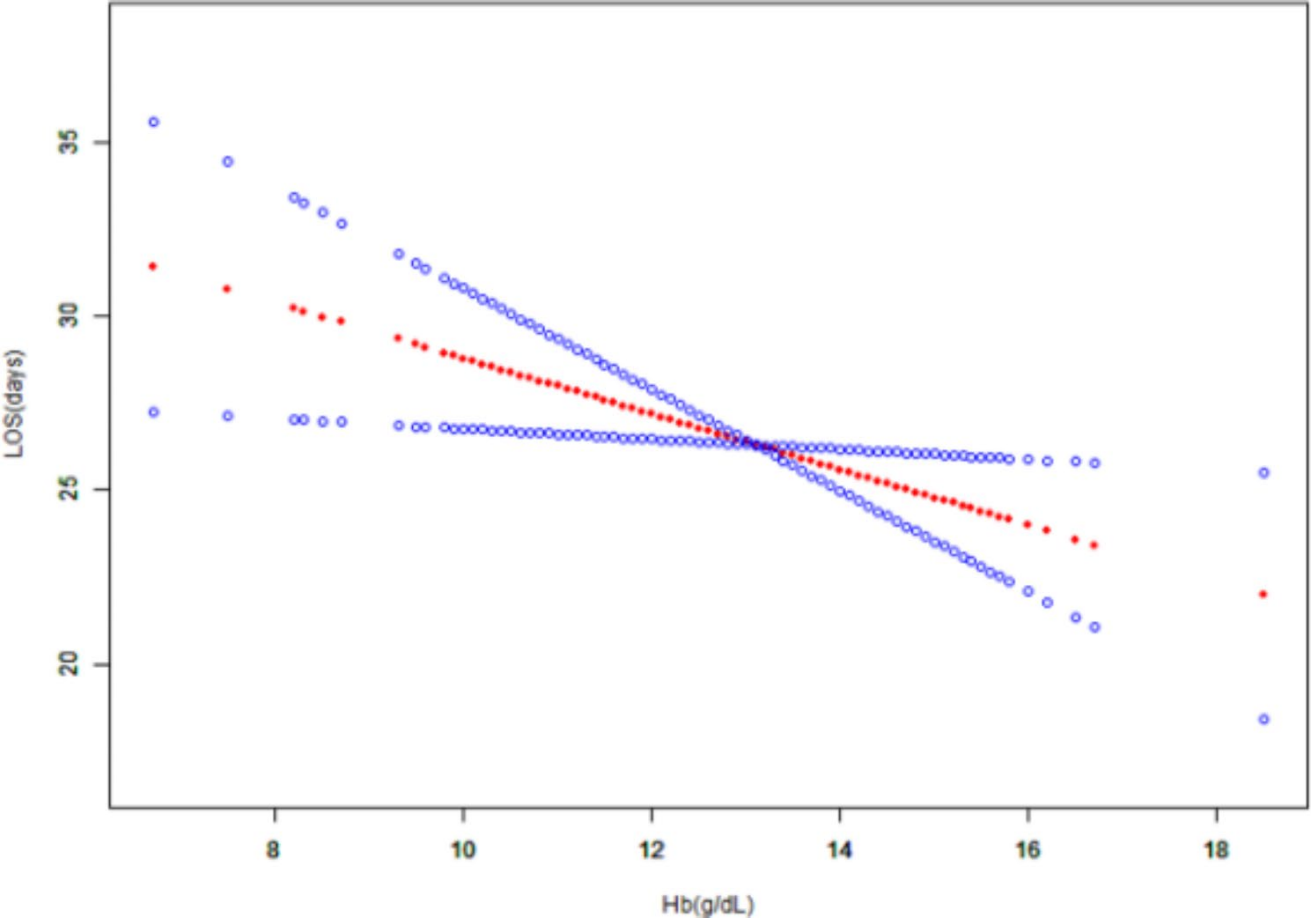
Linear relationship between preoperative Hb and LOS

We examined the non-linear connection between preoperative Hb and LOS to further support the association between preoperative anemia and LOS (Fig. 2). After adjusting for all the covariates included in Table 1’s covariates, a smooth curve and the outcome of a generalized additive model revealed that the relationship between preoperative anemia and LOS was linear. Obviously, we can clearly see that the LOS decreases with the increase in preoperative Hb through Fig. 2. This further suggests that preoperative anemia may prolong a patient’s LOS.

Association between Hb and LOS (After adjustment for other covariates). The smooth curve fit between variables is shown by a solid red line. The 95% confidence interval from the fit is represented by imaginary blue line.

### Subgroup analysis

We used gender; hypertension, age; central nervous system; diabetes; lung disease; heart disease; WBC status; Na^+^ status; K^+^ status; SCr group; PT group; APTT group; preoperative chemotherapy; ASA status; anesthesia method; operation form; blood transfusion group; duration of surgery group; blood loss group; fluid infusion group; postoperative admission to ICU; respiratory failure; lung infection; anastomotic fistula; cardiovascular complication as the stratification variables to observe the trend of effect sizes in these variables (Table 5). We noted that only a small number of interactions were observed based on our a priori specification including: hypertension; WBC; postoperative admission to ICU (all *p*-values for interaction < 0.05).

**Table 5:** Effect size of anemia on LOS (Days) in prespecified and exploratory subgroups in Each Subgroup

Anemia LOS (days)			
	N	β 95%CI	interaction *p*-value
Age, (years) group			0.0570
<60	274	4.79 (1.33, 8.25)	
>=60	373	0.61 (-2.03, 3.25)	
Gender			0.4366
female	146	4.00 (0.02, 7.98)	
male	501	2.02 (-0.44, 4.48)	
Hypertension			0.0245
No	545	1.44 (-0.71, 3.58)	
Yes	102	7.98 (1.33, 14.63)	
Diabetes			0.8676
No	600	2.33 (0.30, 4.36)	
Yes	47	3.00 (-9.53, 15.52)	
Heart disease			0.4683
No	580	2.41 (0.19, 4.64)	
Yes	67	3.30 (-3.15, 9.75)	
Central nervous system			0.7084
No	633	2.43 (0.30, 4.57)	
Yes	14	5.20 (-5.27, 15.67)	
Lung disease			0.8431
No	497	2.27 (-0.07, 4.61)	
Yes	150	2.76 (-1.94, 7.46)	
Abnormal WBC			0.0111
No	579	1.41 (-0.74, 3.57)	
Yes	68	9.99 (1.97, 18.01)	
Abnormal Na^+^			0.2865
No	586	2.10 (-0.10, 4.30)	
Yes	61	5.95 (-1.24, 13.15)	
Abnormal K^+^			0.1797
No	587	1.98 (-0.26, 4.23)	
Yes	60	6.79 (1.11, 12.48)	
SCr, (umol/L) group			0.5045
<100	475	2.09 (-0.28, 4.46)	
>=100	172	3.72 (-0.75, 8.18)	
PT, (s) group			0.9905
<13	632	2.55 (0.40, 4.69)	
>=13	15	2.46 (-3.32, 8.25)	
APTT, (s) group			0.9160
<37	636	2.52 (0.38, 4.66)	
>=37	11	3.39 (-3.97, 10.76)	
Preoperative chemotherapy			0.9437
No	505	2.91 (0.37, 5.46)	
Yes	142	3.11 (-1.77, 7.99)	
ASA ≥ 3			0.7246
No	594	2.28 (0.09, 4.47)	
Yes	53	3.61 (-4.15, 11.37)	
Anesthesia method			0.7903
General + epidural	462	2.25 (-0.05, 4.54)	
General	185	2.87 (-1.71, 7.45)	
Operation form			0.5511
Minimally invasive	300	1.79 (-1.63, 5.22)	
Open	347	3.07 (0.51, 5.62)	
Operation time, (min) group			0.2774
<240	334	3.56 (0.89, 6.24)	
>=240	313	1.25 (-2.01, 4.50)	
Blood loss, (ml) group			0.7595
<200	287	2.84 (-0.42, 6.10)	
>=200	360	2.18 (-0.57, 4.93) 0	
Fluid infusion, (ml) group			0.6503
<2000	39	4.52 (-2.00, 11.05)	
>=2000, < 3000	396	3.02 (0.17, 5.87)	
>=3000	212	1.20 (-2.25, 4.64)	
Preoperative RBC infusion			0.5828
No	520	2.30 (-0.11, 4.71)	
Yes	127	0.86 (-3.72, 5.43)	
Postoperative admission to ICU			0.0101
No	605	1.49 (-0.44, 3.41)	
Yes	42	12.07 (-2.92, 27.05)	
Respiratory failure			0.5127
No	623	2.34 (0.20, 4.49)	
Yes	24	6.02 (-3.92, 15.97)	
Lung infection			0.1027
No	435	1.11 (-0.83, 3.06)	
Yes	212	4.77 (-0.12, 9.67)	
Anastomotic fistula			0.8244
No	605	2.69 (1.09, 4.28)	
Yes	42	3.54 (-12.96, 20.03)	
Cardiovascular complication			0.8043
No	614	2.29 (0.19, 4.38)	
Yes	33	5.76 (-7.50, 19.02)	

Abbreviations: LOS, length of stay; WBC,white blood cells; Na^+^, Serum sodium ion; K^+^, Serum potassium ion; SCr, Serum creatinine; PT, prothrombin time; APTT, activated partial thromboplastin time; ASA, American Society of Anesthesiologist; RBC, red blood cell; ICU, Intensive care unti

## Discussion

Preoperative anemia was common among patients having radical esophagectomy at our facility, with a prevalence of 33.6%, according to a retrospective cohort analysis of 647 patients, similar to the previously reported prevalence of anemia in digestive malignancies (27.5%) [10]. Similarly, Caro JJ and Tesarova P et al. reported that anemia is a common disease in cancer patients, with anemia occurring in about 30% of cancer patients [6, 7]. According to previous studies, blood marrow deficiency or malnutrition, bleeding from tumor sites, metastasis, catabolism in tumor patients, and relative deficiency of erythropoietin may be involved in the pathogenesis of anemia [21]. The role of multimodal therapy for oesophageal cancer remains controversial, while at the same time, the use of neoadjuvant therapy is becoming increasingly common. In this study, the proportion of preoperative adjuvant chemotherapy was as high as 21.95%. Nearly half of the patients in the anemia group had preoperative chemotherapy, which was significantly higher than that in the non-preoperative anemia group. This may be a side effect of chemotherapy drugs, which damage hematopoietic stem cells and cause anemia. In addition, platinum drugs can cause kidney damage, which can reduce the secretion of erythropoietin (EPO) and further aggravate anemia. In our study, patients in the preoperative anemia group had a higher blood transfusion rate. These results are similar to previous studies by Musallam KM et al. and Abdullah, H.R. et al. [22, 23]. The International Agency for Research on Cancer estimates that males are two to four times more likely than women to get esophageal cancer [24]. In this study, the prevalence was 77.43% in men and 22.57% in women, consistent with previous studies. The operation for esophageal cancer is difficult and the recovery is slow.

Oesophageal cancer is the eighth most prevalent cancer worldwide and the sixth most deadly malignancy [1]. As a common malignant tumor of the digestive tract, esophageal cancer has become an important disease endangering human health worldwide. For the sole curative treatment of resectable oesophageal cancer, surgical resection with radical lymphadenectomy, often after neoadjuvant chemotherapy or chemoradiotherapy, is recommended [2–4]. However, the vast majority of patients with esophageal cancer spend a long time in the hospital because of poor preoperative basic conditions, great surgical difficulties, and slow postoperative recovery.

Through the Cox univariate model in Table 2, we found that the patient had preoperative anemia; age; hypertension; diabetes; preoperative hemoglobin; operation time ≥ 240 min; perioperative red blood cell infusion; postoperative admission to ICU; respiratory failure; pulmonary infection; anastomotic leakage, which is closely related to the prolongation of LOS. It is proven that they are risk factors for prolonged LOS in patients with esophageal cancer.

We discovered in the current research that individuals with preoperative anemia who underwent radical esophageal cancer surgery lasted 2.04 days longer than patients without preoperative anemia After adjustment for potential risk factors associated with LOS, that is to say, preoperative anemia is positively associated with LOS after adjusting for other covariates. Confounding factors include age; central nervous system; gender; hypertension; heart disease; lung disease; diabetes; WBC; Na^+^; K^+^; SCr; PT; APTT; preoperative chemotherapy; ASA; anesthesia; operation form; blood transfusion; operation time; blood loss; fluid infusion; postoperative admission to ICU; respiratory failure; lung infection; anastomotic fistula; cardiovascular complication. With the use of subgroup analysis, we discovered that the only factors that may affect the correlation between preoperative anemia and LOS were hypertension, WBC, and postoperative ICU hospitalization. After adjusting for the above covariables, the three models’ predictions for preoperative anemia and LOS were steady and trustworthy.

According to earlier research, individuals with preoperative anemia often stay in the hospital longer. Poon, E. et al., identified poor surgical outcomes in patients with preoperative anemia following intestinal surgery, including increased blood transfusion rates and longer hospital stays [9]. In addition, Sanoufa, M. et al. also found that preoperative anemia significantly prolonged the hospital stay of patients undergoing spinal surgery [11]. Similarly, in a large prospective observational study in South Africa, Marsicano, D. et al. conducted a study on patients receiving non-cardiac and non-obstetric inpatient surgeries in 50 hospitals around the world and found that patients with preoperative anemia had significantly longer hospital stays than patients with preoperative normal hemoglobin [12]. In addition, some studies have pointed out that anemia patients will increase the chance of blood transfusion, leading to overall postoperative complications and wound infection rate, thus affecting patients’ early recovery and prolonging hospital stay [13, 14]. Our results proved that patients undergoing radical esophageal cancer surgery with preoperative anemia had a longer hospitalization for preoperative anemia than patients without preoperative anemia. In previous studies, the independent relationship between preoperative anemia and LOS was basically analyzed. The difference between our study and previous studies is that we also made a multiple regression model for the quantified preoperative Hb and LOS and adjusted all variables in Table 1. The results of the three models are very stable: with the increase of preoperative Hb, LOS will be shortened accordingly. When we defined anemia as HB < 13 (g / dl) regardless of the role of gender, we obtained results similar to those in Table 3, indicating that whether according to the WHO anemia grade or the anemia grade regardless of gender, the results show that preoperative anemia can prolong LOS in patients with esophageal cancer. Through the sliding curve and generalized additive model, after adjusting for all covariates in Table 1, it can be intuitively observed that there is a very obvious linear relationship between preoperative Hb and LOS, which further proves the relationship between preoperative anemia and LOS.

Improving the quality of medical care while also improving hospital efficiency is a priority for our healthcare system. Length of hospital stay is often used to evaluate hospital quality in surgery, and shortening the length of hospital stay is of great significance to reduce overall healthcare costs [25]. Similarly, Vendittoli P et al. point out that reducing the cost of care would reduce the financial pressure on health insurance, save health care and health resources and lower the cost of care for patients [26]. Lovald, S.T. et al. suggested that shorter hospital stays might improve patients’ functioning and quality of life [27]. In addition, Husted H et al. argued that patients with shorter hospital stays tend to be more satisfied with surgery than patients with longer hospital stays [28]. Thus, the index of the length of stay can not only reflect the length of stay from hospitalization to discharge of a patient but also indirectly reflect the hospital’s medical service capacity, service quality, and management of medical treatment and skill level. Shortening LOS, accelerating the operation of beds, rational utilization of medical resources, and improving the rational utilization of medical resources in the health system play a great role. Therefore, it is becoming increasingly necessary to identify preoperative risk factors that influence the patient’s length of stay. We know that esophageal cancer surgery is difficult, recovery is slow, and hospital stay is long, so it is important to explore more factors related to the hospital stay. In fact, the search for a relationship between radical resection of esophageal cancer and length of hospital stay has not stopped. Bundred, J.R. et al. pointed out that minimally invasive esophagectomy can significantly shorten the length of hospital stay compared with open surgery [29]. Ethan Y Song et al. analyzed a database of 1031 cases of esophagectomy in a US hospital and found that patients aged 80 and older had an average increase of 2.4 days in hospital stay compared with those aged 80 and younger [30]. In addition, Dent B et al. found that postoperative anastomotic leakage can prolong the duration of intensive care and the total hospital stay of patients [31]. However, there have been few reports of whether preoperative comorbid anemia in patients with esophageal cancer affects hospital stay. In our study, patients with preoperative anemia before surgery had a relatively long hospital stay. This may be because preoperative anemia increases the perioperative blood transfusion rate and the incidence of postoperative adverse events in patients, which needs to be further confirmed by subsequent studies. Preoperative adjustment of hemoglobin may benefit the early recovery of patients and shorten the length of hospital stay, but this needs to be further confirmed by subsequent studies.

It is also worth discussing in the study that Table 1 shows chemotherapy patients have more preoperative anemia. However, it indicates that chemotherapy is not significantly associated with increased LOS, but anemia is associated with increased LOS. While chemotherapy can cause anemia, it may not be the primary contributor to increased LOS in surgical patients. On the other hand, anemia itself can increase the risk of postoperative complications, impair tissue oxygenation, and delay wound healing, all of which can prolong hospital stay [32]. Therefore, managing anemia preoperatively may help mitigate these risks and improve patient outcomes. Further research is needed to elucidate the mechanisms linking anemia and LOS in surgical patients, and to identify effective interventions to optimize perioperative care for this population [22].

Our research has the following advantages: (1) Independent analysis of the association between preoperative anemia in esophageal cancer patients with the radical disease and LOS; (2) Independent analysis of the relationship between preoperative Hb in patients with esophageal cancer radical disease and LOS; (3) Enough variables were adjusted to avoid the influence of confounding factors on the research results; (4) The results were trustworthy and steady, with little missing data. This could be a result of the necessary, prospective electronic collection of our clinical data, such as preoperative factors, during the standard preoperative anesthetic examination; (5) Since this was an observational study, various confounders could have affected the results; nonetheless, we strictly applied a statistical correction to reduce residual variables; (6) Reasonably solid conclusions were drawn for various study subgroups; (7) We made the smooth curve and generalized additive model of Hb and LOS before the operation to make the results more intuitive and visible.

In our study, we took steps to mitigate some of these limitations by carefully selecting our study population and controlling for known confounders in our statistical analyses. We also conducted sensitivity analyses to test the robustness of our findings. Nonetheless, we recognize that our results should be interpreted with caution and cannot be used to establish causality.

Our study has the following drawbacks: (1) The subjects were patients undergoing radical resection for esophageal cancer; therefore, these results do not apply to patients undergoing other types of surgery; (2) People who underwent further procedures after being excluded from unanticipated subsequent surgery cannot use the study’s findings; (3) There may be variations in treatment approaches, the medical staff, etc. because this work was a retrospective analysis and the data used were clinical data from a single center. For example, because our data were collected on perioperative blood transfusions of patients before, during, and after surgery, however, due to the limitations of the information system, we cannot obtain the anemia treatment strategy for every patient. There may have been some influencing factors that were left out of the research, such as baseline characteristics (albumin, sarcopenia, BMI, patient income, medical costs, and health insurance), as well as other factors that may have had an effect on the outcomes. We don’t know what the attending physician used to treat anemia (iron or blood transfusion, etc.) before the operation began. During the operation stage, each doctor in charge of anesthesia may have different blood transfusion strategies depending on the needs of the operation and the patient’s own situation. The treatment options for anemia after esophageal cancer surgery are also unknown. More data is needed for further study on whether the treatment plan for preoperative anemia will affect the results of our study. In addition, as our data is from 2010 to 2020, the proficiency of surgeons may vary with the passage of time, or the level of different surgeons may vary, which needs to be discussed in further detail.

It is challenging to establish a causal link between preoperative anemia and unfavorable outcomes because the study was observational in nature. However, this study provides insight into the association between preoperative anemia and LOS in patients undergoing radical esophageal cancer resection.

## Conclusion

Our research demonstrated that preoperative anemia is relatively common in Chinese patients undergoing radical esophageal cancer resection and is independently related to a longer LOS. The patient’s hemoglobin level needs to be monitored during the preoperative phase. Future research must validate this finding.

## Data Availability

The datasets generated during and analyzed in the current study are not publicly available due to institutional restrictions but are available from the corresponding author on reasonable request.
